# Identifying and Qualifying Deviant Cases in Clusters of Sequences: The Why and The How

**DOI:** 10.1007/s10680-023-09682-3

**Published:** 2023-12-19

**Authors:** Raffaella Piccarreta, Emanuela Struffolino

**Affiliations:** 1 Department of Decision Sciences, “Carlo F. Dondena” Centre for Research on Social Dynamics, BIDSA (Bocconi Institute for Data Science and Analytics), Bocconi University, via Guglielmo Röntgen 1, 20136 Milan, Italy; 2 Department of Social and Political Sciences, University of Milan, via Conservatorio 7, 20122 Milan, Italy

**Keywords:** Cluster analysis, Sequence analysis, Visualisation, Index plot, Flagged index plot

## Abstract

Sequence analysis is employed in different fields—e.g., demography, sociology, and political sciences—to describe longitudinal processes represented as sequences of categorical states. In many applications, sequences are clustered to identify relevant *types,* which reflect the different empirical realisations of the temporal process under study. We explore criteria to inspect internal cluster composition and to detect *deviant* sequences, that is, cases characterised by rare patterns or outliers that might compromise cluster homogeneity. We also introduce tools to visualise and distinguish the features of regular and deviant cases. Our proposals offer a more accurate and granular description of the data structure, by identifying—besides the most typical types—peculiar sequences that might be interesting from a substantive and theoretical point of view. This analysis could be very useful in applications where—under the assumption of within homogeneity—clusters are used as outcome or explanatory variables in regressions. We demonstrate the added value of our proposal in a motivating application from life-course socio-demography, focusing on Italian women’s employment trajectories and on their link with their mothers’ participation in the labour market across geographical areas.

## Introduction

Sequence analysis (SA; see Liao et al., 2022; Piccarreta & Studer, 2019; Raab & Struffolino, 2022 for an introduction) is a collection of tools used in social science to simultaneously describe the timing, sequencing, and *quantum* of processes that are represented by sequences of events (*states*) experienced over a specific period and observed at regular intervals. Such sequences are regarded as meaningful units of analysis and studied in their entirety. Because longitudinal data have recently become widely available, SA has been extensively used in demography and life-course research and beyond: Its versatility in analysing the evolution of processes over time has been proven by applications in other fields (social policy analysis, democratisation research, electoral participation studies, historical sociology, developmental psychology, mobility and time use research, and study of cultural processes).

In many applications, sequences are clustered to reveal relevant *types,* reflecting the different structural features of the temporal evolution of a given process. An important issue concerns the assessment of within-cluster homogeneity, the relevance of the identified types, and the detection of sequences with peculiar or uncommon features. In this Research Note, we contribute to the literature by proposing criteria that can be used to inspect clusters internal composition and to identify *deviant* cases in clusters and therefore in data. Further, we introduce tools to visualise and suitably describe the features of deviant sequences, thus enhancing the description of the data structure.

Clusters typically include some sequences that deviate—sometimes substantially—from the type that their cluster supposedly represents. We distinguish between two types of deviant cases. *Structurally peculiar* sequences differ from their cluster’s core but are nonetheless similar to each other; they are hence one of the relatively rare types of sequence that could be isolated in dedicated clusters by increasing the number of clusters (even if this might be accomplished only by very detailed and fragmented partitions). By contrast, *outlying* sequences have very peculiar features and are therefore highly different from *all* the others in their cluster.

Identifying the different deviant trajectories and their characteristics is relevant for several reasons. First, it offers a better description of the types—meaning that deviating sequences cannot influence substantive interpretations of the typical patterns—and it informs a more conscious and cautious interpretation of the substantive results. Second, it permits a detailed analysis of within-cluster heterogeneity: If clusters include many structurally peculiar sequences, this might indicate that the chosen partition failed to identify all the substantively/theoretically relevant types in the data. Further, deviant sequences might be interesting in their own right from a substantive and/or theoretical viewpoint. Indeed, clustering algorithms typically—and with good reason—prioritise the identification of the most typical patterns, and marginal clusters or outliers can be difficult to identify.

Efforts to detect deviant sequences may be particularly beneficial when clusters enter subsequent regression analyses as dependent or independent variables. This is only suitable if clusters are homogeneous, so that sequences clustered together share common features and are well represented by the same *type*. Moreover, deviant sequences might also differ more from others in their clusters with respect to covariate levels when a strong association exists between sequences and covariates: this could influence and bias the regression’s results.

To illustrate our procedure and demonstrate its added value, we analyse Italian women’s employment trajectories and their link with the respondents’ maternal participation in the labour market across geographical areas.

## Identifying Deviant Sequences

In the following, we introduce criteria to identify and analyse *deviant* sequences.^1^ These differ from others in their cluster with respect to the most relevant characteristics of the process signified by the cluster itself or, in other words, by the *type* that summarises the features of sequences allocated to the cluster.

### Identifying Cluster-Dissimilar Sequences

A first intuitive approach to detecting deviant sequences relates to the extent of their dissimilarity from their cluster. For the generic $i$th sequence, this can be evaluated based on its average dissimilarity from all the others in its cluster, $a_i$, or on its dissimilarity, $m_i ,$ from its cluster’s representative sequence. Here, we look at the medoid, that is, the case with the minimal average distance from all the others. Thus, for a sequence $s_i$ in a cluster $C_g$ including $n_g$ cases summarised by the medoid $\tilde{s}_g$ we consider:

$$\begin{aligned} & a_i = \sum \limits_{j \in C_g ,j \ne i} d(s_i ,s_j ){/}(n_g - 1) \\ & m_i = d(s_i ,\tilde{s}_g ), \\ \end{aligned}$$

where $d(s_i ,s_j )$ indicates the (properly measured) dissimilarity between two sequences. To evaluate individual deviations in relative terms, we compare them with the means calculated on all the cases, $\overline{a} = \sum_i {(a_i {/}n)}$ and $\overline{m} = \sum_i {(m_i {/}n)}$. As for all the criteria proposed to flag extremes, deviations could be compared to a threshold, which may coincide with the percentile of the deviations (chosen based on the “expected” proportion of extreme cases). Since thresholds will (or should) also depend on the amount of heterogeneity in data, we adopt an exploratory approach and suggest analysing which sequences are identified as dissimilar from their cluster as the threshold varies.

### Identification of Noisy Sequences

Another approach to detecting deviant sequences is based on the criterion used in the DBSCAN clustering algorithm (Ester et al., 1996) to classify cases into core, border and noise. Specifically, core cases are those in ‘dense’ regions that have a number of neighbours (cases located within a pre-defined distance from them) that exceed a specific threshold. Border cases are non-core cases located in the neighbourhood of a core case. Isolated cases that are neither core nor border cases are defined as noise. These definitions require a specification of the radius of the neighbourhood and the minimum number of cases it should include. We adopt a data-driven approach, and consider the dissimilarity of each sequence from its 5th nearest neighbour (NN) in its cluster, $d_i^{{\text{NN}}5}$, and a high percentile—e.g., the 95th, $\overline{d}_{0.95}^{{\text{NN}}5}$—of the distribution of the $d_i^{{\text{NN}}5}$’s in the entire sample. Therefore, 95% of the sequences in the sample have at least 5 NN (in their cluster) within a distance lower than or equal to $\overline{d}_{0.95}^{{\text{NN}}5}$. We define core sequences as those having at least 5 sequences in their cluster within a distance $\overline{d}_{0.95}^{{\text{NN}}5}$, and border and noise sequences accordingly. Researchers need to choose the number of NN and the percentile of the distances to use as a threshold. Nonetheless, because case classification and neighbourhood diameter depend on the behaviour of *all* the cases—due to the reliance on percentiles—the chosen number of NN has a limited impact on the identification of noise cases.

### Visualising Deviant Sequences

Sequences and clusters are usually explored using the *sequence index plot* (Scherer, 2001), where each sequence is represented by a set of horizontally stacked bars coloured based on the state visited at each time point. To enhance visualisation, individual sequences are arranged on the vertical axis based on their similarity (see Piccarreta & Lior, 2010). This plot might fail to accurately describe the patterns in large sets of data, because sequences are over-plotted to make them fit into the graphic window: visualising and drawing substantive conclusions on clusters’ features can therefore be difficult. Alternative plots introduced in the literature (Fasang & Liao, 2014; Gabadinho et al., 2011; Müller et al., 2008; Piccarreta, 2012) limit over-plotting by only displaying sequences representative of their clusters, thus excluding the deviant sequences we are mostly interested in from the plot.

To explore the features of both regular and deviant clusters’ sequences, we propose to associate each cluster with a set of *flagged index plots*, one dedicated to regular cases and the other/s dedicated to sequences deviating according to one (or more) criterion or according to the severity of the deviation. This enables a close inspection of irregular sequences.

## Illustrative Application

The increase in women’s labour market participation from the 1950/1960s is one of the most crucial changes in the post-war Italian labour market, although female participation remains among the lowest in the EU-27 (around 50% in 2019; Eurostat, 2020), with large disparities across the country. We focus on the employment trajectories of Italian women who transitioned from school to work between late 1970s and early 1990s. Different cohorts were differently exposed to labour market deregulation, but the ‘access’ to a stable employment trajectory remained associated with individual characteristics and parental background (Raitano & Vona, 2018; Struffolino & Raitano, 2020). Besides the structural constraints discouraging women—and particularly mothers—from participating in the labour market (e.g., lack of accessible childcare, residual parental leave for fathers, persistent wage gaps), maternal employment has proved to be a driver of women’s employment and intergenerational mobility, over and above parental education. Mothers’ employment can influence their daughters’ behaviour via social learning—by providing actual behavioural examples (Bandura, 1977), and by changing gender attitudes (Moen et al., 1997). Empirical evidence on single countries (e.g., Di Pietro & Urwin, 2003 for Italy) and cross-country analyses (e.g., McGinn et al., 2019) has shown that adult daughters of employed mothers are more likely to be employed. These studies focus on point-in-time responses, for example, the respondent’s employment status at age 35. Here, we instead consider a long time span over individuals’ life courses to identify specific forms of inequality in labour market trajectories.

### Data and Methods

We used data from the ‘Multi-purpose Survey on Households: Families and Social Subjects’ carried out in 2009 by the Italian National Statistical Office (ISTAT) and focused on 4323 women born between 1959 and 1974 whose monthly work activities were tracked from age 16 to 35. We distinguish between the following states: education (Edu),^2^ joblessness (Jless, including both unemployment and inactivity), full-time (FT), and part-time (PT) work, further broken down according to the type of contract: permanent (Perm), temporary (Temp), self-employment (Self), or dependent self-employment (DSelf).^3^

First, we applied cluster analysis to identify distinctive employment trajectories. We assessed dissimilarities between sequences using OMA (Abbott, 1990; Abbott & Forrest, 1986) with substitution costs between two states being inversely proportional to the transition frequencies from one state to the other, and insertion-deletion costs equal to 1. We extracted clusters using partitioning around medoids (PAM, Kaufman & Rousseeuw, 2005) and the Ward’s agglomerative algorithms and compared the quality of partitions with a varying number of clusters using a battery of criteria (point biserial correlation, Hubert’s gamma, Hubert’s C coefficient, average silhouette width, pseudo *R*^2^ and pseudo *F* statistic; Hennig & Liao, 2010; Studer, 2013). Most of the criteria (Fig. 3, Appendix) pointed to 4 clusters and indicated that the PAM algorithm performed better.

We identified deviant cases based on three criteria: (a) *average* dissimilarity from other cases in their cluster more than 1.5 times the general average; (b) *dissimilarity* from own cluster’s *medoid* more than 1.5 times the general average; and (c) identification as *noisy* based on the $\overline{d}_{0.95}^{{\text{NN}}5}$-criterion.

We analysed cluster composition by distinguishing between regular and deviant sequences. We then used multinomial regression models to relate trajectories—and specifically cluster membership—to the interaction between maternal employment status (employed or unemployed/inactive) when the respondent was 15 years old and the geographical macro-area of residence (North-East, North-West, Centre, South, or Islands) at the time of interview, controlling for birth cohort (1959–1964, 1965–1969, or 1970–1974), highest parental education level (no education, primary education, at least lower secondary education), and whether at least one parent had tertiary education (see Table 1 in the Appendix for the covariate distribution in the sample). Specifically, we analysed whether and to what extent including or excluding the identified deviant sequences affected the regression’s results.^4^

### Results

**Fig. 1:**
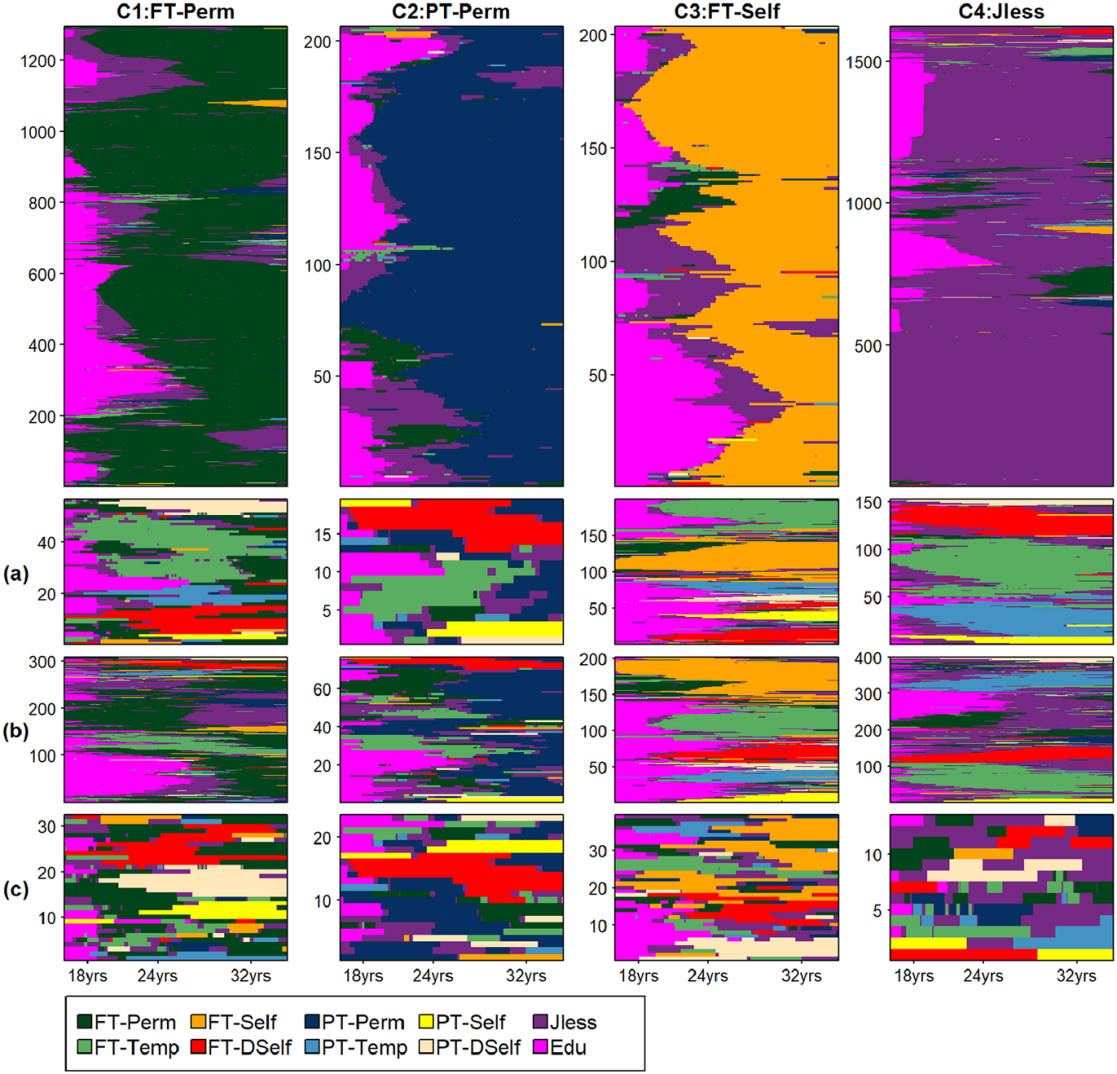
Flagged index plots for the 4-clusters partition extracted using PAM clustering algorithm. For each cluster (column), the top plot reports cases flagged as regular by all the criteria used to identify deviant cases. For each cluster, we report subplots displaying sequences flagged as deviant, being dissimilar from their cluster [according to their average dissimilarity from their cluster—(**a**)—or to their dissimilarity from the cluster’s medoid—(**b**)], or being identified as noisy—(**c**). In each plot, cases are arranged on the vertical axis based on the *Traveling salesperson problem solver* (TSP, Gutin & Punnen, 2007) seriation algorithm (Hahsler & Hornik, 2011; Hahsler et al., 2008), starting from a randomly chosen case finds the shortest path (in terms of dissimilarities) to connect all the cases. *Source*: Multi-purpose Survey on Households: Families and Social Subjects 2009. (Color figure online)

Figure 1 displays the *flagged index plots* for the four-cluster partition. For each cluster (column), the top plot reports cases flagged as regular by all the three criteria (possibly overlapping) used to identify deviant cases. Note that the clusters’ regular sequences exhibit very distinguishable features, with long-term permanence in FT-Perm (C1), PT-Perm (C2), FT-Self (C3), and Jless (C4). People enter these dominant states after a variable period of education: half of the cases in C4 left school before 18. In contrast, C3 includes a high proportion of women who prolonged education, possibly experiencing relatively long joblessness before becoming self-employed.

Besides the plots displaying regular sequences, for each cluster we report subplots displaying sequences *flagged* as deviant, being dissimilar from their cluster (according to their *average* dissimilarity from their cluster or to their *dissimilarity from* the cluster’s *medoid*), or being identified as noisy*.* The sets of cases dissimilar from their cluster (Fig. 1, rows a and b) mostly include structurally peculiar sequences dominated by employment arrangements less diffused among Italian women in the relevant age range, in particular FT and PT combined with Temp and DSelf. Despite their common features, such sequences are scattered among the four clusters, mainly based on the length of education.

The sets of sequences flagged as noisy (Fig. 1, row c) include sequences sharing similar—even if atypical—patterns that are dominated by extremely rare states (particularly, PT-DSelf) as well as turbulent sequences with very uncommon characteristics (e.g., high numbers of transitions or rare transitions). These cases do not share common or relevant (in terms of frequency) patterns and can therefore be regarded as outliers with irregular or unstructured deviations from the cluster.

While some deviation from the regular core sequences is expected, because several factors affect individuals’ behaviours (e.g., preferences, resources, constraints), not all the degrees of structural deviations are acceptable. Thus, the allocation to clusters of sequences with structural differences from the core ones should be justified/explained based on some substantive considerations. Indeed, this might affect the substantive interpretation of the typology deduced from the clusters. Regarding the outliers, researchers should consider if they can be regarded as random deviations from the cluster types or rather as sequences that do not belong to clusters.

Identifying peculiar patterns enables researchers to describe the data in more detail; moreover, deviant sequences might be interesting in their own right from a substantive and/or theoretical point of view. However, it is not necessarily easy or possible to identify such sequences by increasing the number of clusters, since clustering algorithms typically—and reasonably—prioritise the identification of the most typical patterns.

Indeed, we also explored higher-order partitions and in particular (based on the criteria used to assess clusters’ quality) the PAM nine-clusters solution (Fig. 4, Appendix). While identifying two clusters, including the structurally peculiar sequences dominated by FT-Temp and by prolonged education, respectively, this solution offers a more accurate description of the sequences dominated by the most frequent states, namely FT-Perm and JLess; the less frequent deviant sequences are not detected and isolated. Interestingly, we applied the PAM algorithm to group the sequences flagged as deviant in the four-cluster solution (Fig. 1, rows a, b, and c), and extracted 11 clusters (Fig. 6 and Table 2, Appendix). Some of these ‘deviant’ clusters remained highly heterogeneous but we detected some small clusters of trajectories dominated by non-standard employment, which could not be identified even by increasing the number of clusters (for example, 15 as displayed in Fig. 5, Appendix) extracted from the entire set of cases. Thus, in addition to being able to explore and address within-cluster heterogeneity, our suggested procedure also enabled an efficient analysis and characterisation of both regular types and rare or less frequent types or sub-types.

As mentioned above, the identification of deviant sequences can be particularly important when clusters are related to covariates via multinomial logistic regression. Indeed, if an actual relation exists between covariates and clusters, structurally deviant sequences might be characterised by structural differences in their covariates’ values, and this might affect the significance and/or the magnitude of regression coefficients. For example, if deviant sequences that possess the opposite covariate characteristics to those of the core sequences are included in a cluster, this can lower the significance of coefficients; by contrast, deviant sequences with extreme covariate values can magnify the relevance of a covariate that is not related to given clusters.

**Fig. 2:**
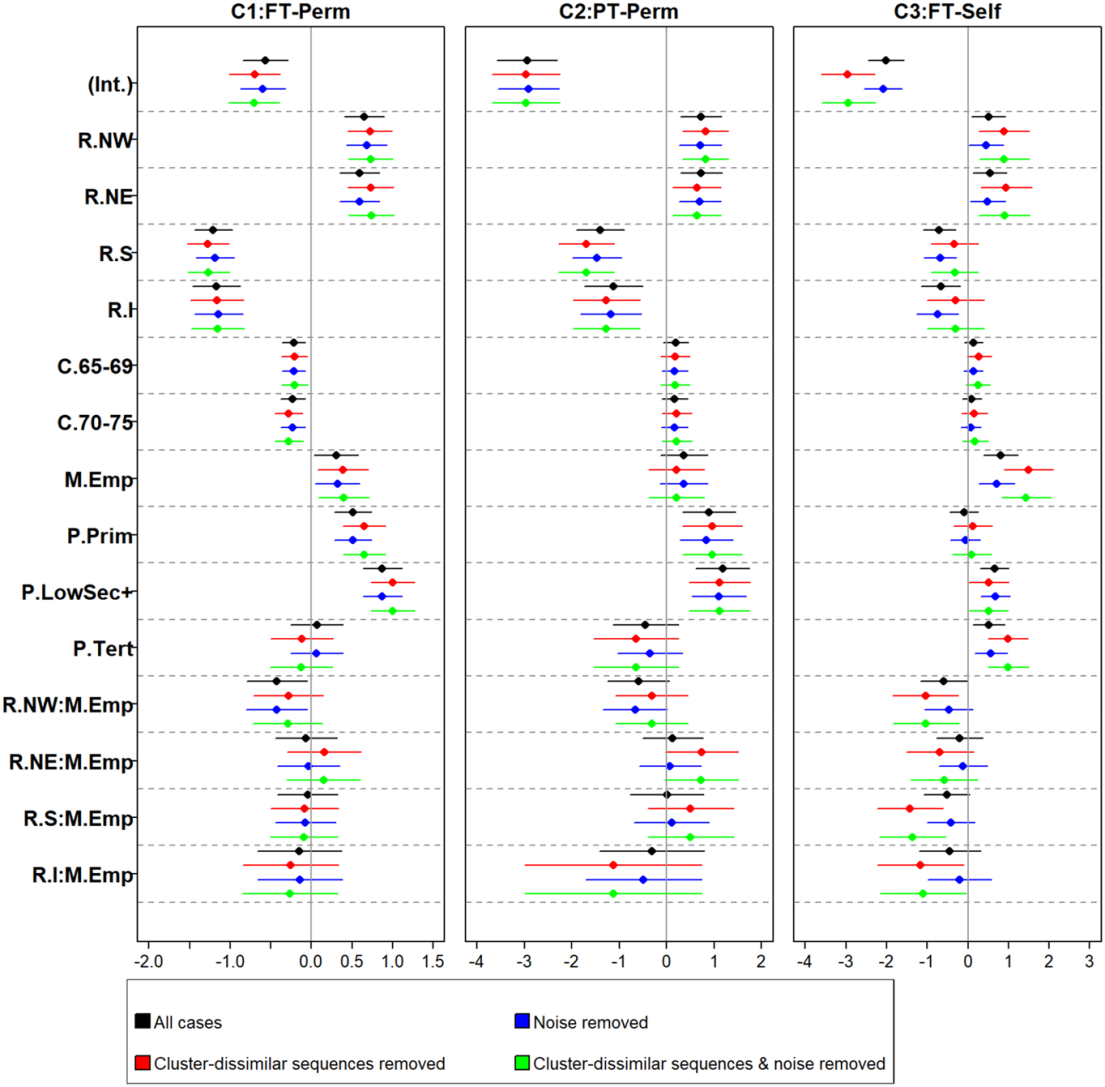
Beta-coefficients of a multinomial logistic regression model for cluster membership (reference category: C4:JLess) and 95% confidence intervals. Regression is first applied to the entire sample (black) and then to the sub-samples obtained by filtering out different types of deviant sequences: (red) sequences dissimilar from their cluster—i.e. sequences with an average dissimilarity from the others (in their cluster) or from their cluster’s medoid higher than 1.5 times the corresponding general average (blue) noisy sequences identified based on $\overline{d}_{0.95}^{{\text{NN}}5}$ (green) both sequences dissimilar from their cluster and noisy sequence. *Source*: Multi-purpose Survey on Households: Families and Social Subjects 2009. Legend for covariates: Int. = intercept; macro-area of residence at the age of 15: North-East (R.NE), North-West (R.NW), Centre (reference), South (R.S), and Islands (R.I); birth cohort: 1959–1964 (reference), 1965–1969 (C.65–69), 1970–1975 (C.70–75); working status of the mother when the respondent was 15: employed (M.Emp), not employed (reference); highest parental education level: no education (reference), primary education (P.Prim), at least lower secondary education (P.LowSec+); at least one parent with tertiary education (dummy variable, P.Tert). (Color figure online)

**Fig. 3:**
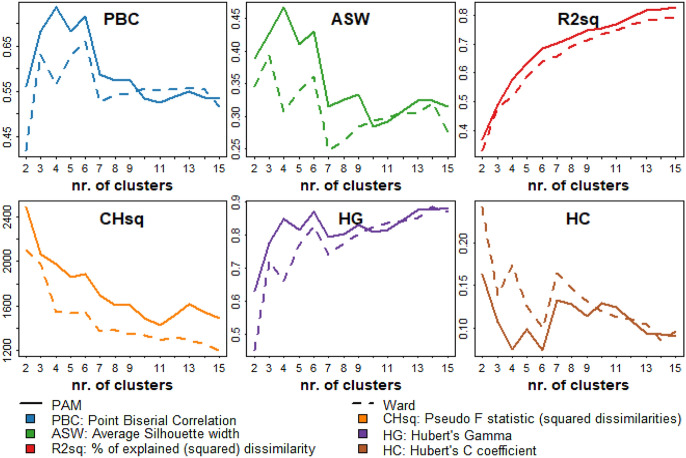
Quality of partitions extracted using Ward’s agglomerative algorithm and the Partitioning Around Medoids (PAM) for a varying number of clusters. Optimal solutions are those corresponding to maximal values, with the exception of the Hubert’s C coefficient that should be minimised. *Source*: Multi-purpose Survey on Household and Social Subjects 2009. (Color figure online)

**Fig. 4:**
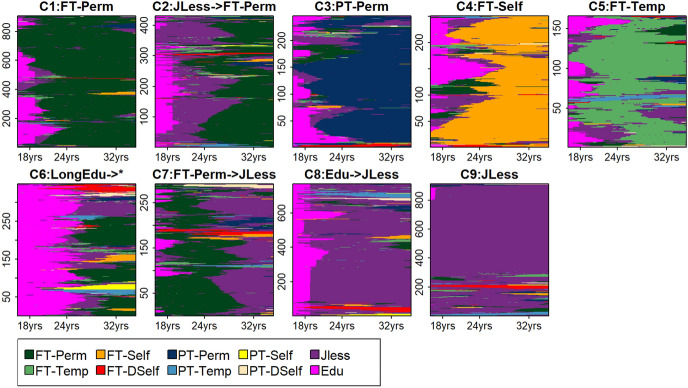
Index plots for the 9-cluster partition extracted using PAM clustering algorithm. *Source*: Multi-purpose Survey on Household and Social Subjects 2009. (Color figure online)

**Fig. 5:**
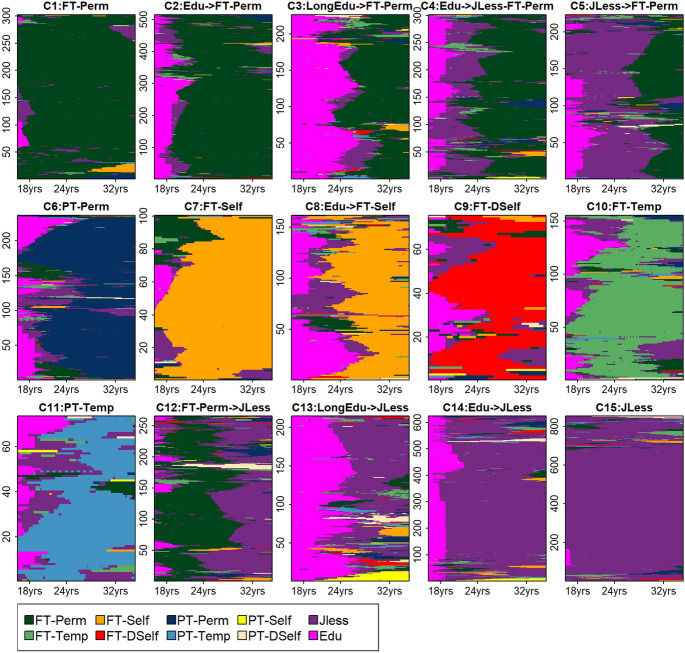
Index plots for the 15-clusters partition extracted by using PAM clustering algorithm. *Source*: Multi-purpose Survey on Household and Social Subjects 2009. (Color figure online)

**Fig. 6:**
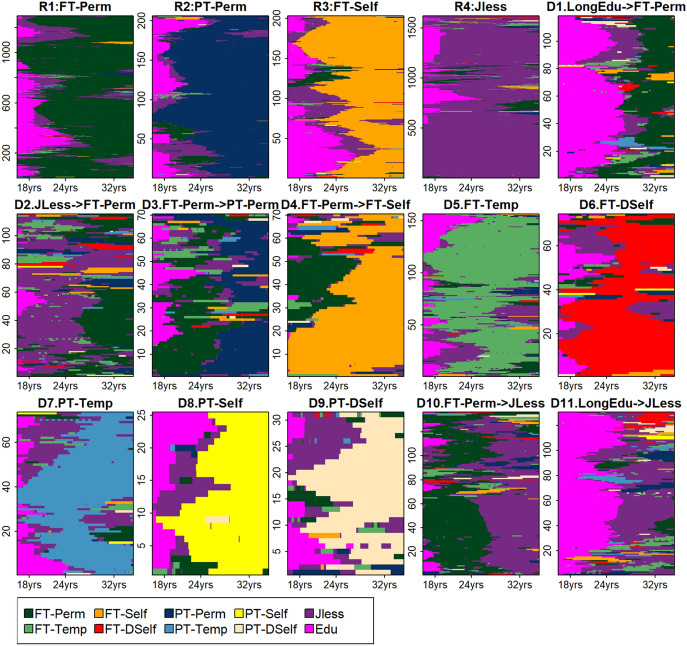
Index plots of the cases identified as *regular* in our 4-clusters partition (R1–R4) and of the 11-clusters (D1–D11) extracted by applying PAM clustering algorithm to the cases flagged as *deviant* in the 4-clusters solution. *Source*: Multi-purpose Survey on Household and Social Subjects 2009. (Color figure online)

**Fig. 7:**
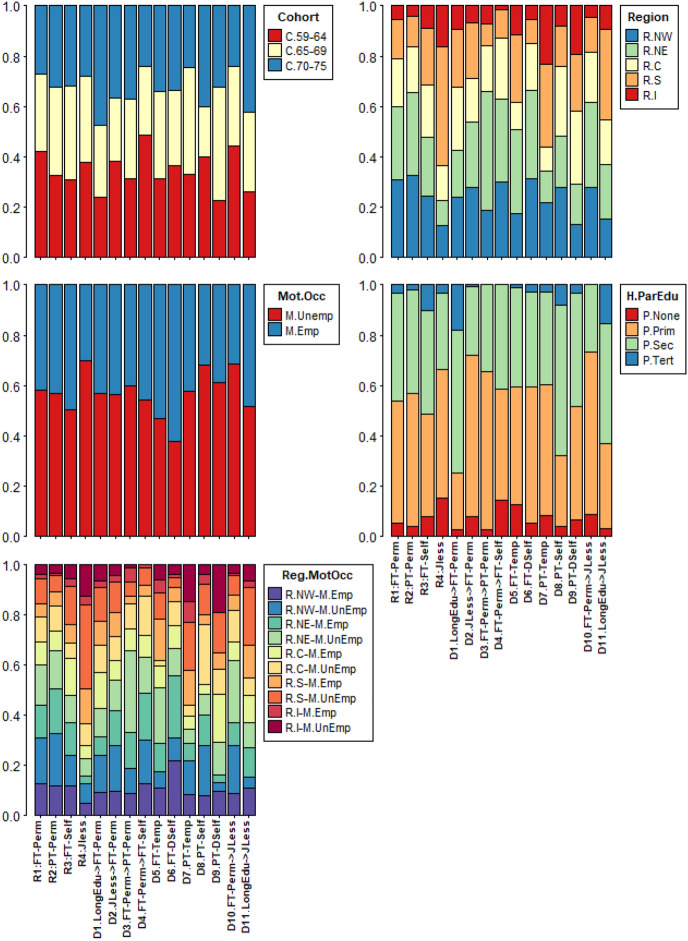
Distributions of the (categorical) covariates included in the regression model within the clusters of regular and deviant cases. Cohort: birth cohort (C.59–64:1959–1964; C.65–69: 1965–1969; C.70–75:1970–1975); Region: region of residence (R.NW: North-West; R.NE: North-East; R.C: Center: R.S: South; R.I: Islands); Mot.Occ: the mother was in employment (Emp) or unemployed (UnEmp) when the respondent was 15 years-old; H.ParEdu: highest parental education level; Reg.MorOcc: interaction between region of residence and mother’s position in the labour market. *Source*: Multi-purpose Survey on Households: Families and Social Subjects 2009. (Color figure online)

Drawing on our data, Fig. 2 reports the coefficients of the models estimated based on the entire sample and on the sub-samples obtained by filtering out deviant sequences identified using different criteria. We focused on the coefficients for maternal employment (M.empl). When we estimated the model using the entire sample (in black), having had a mother who worked when the respondent was 15 increased the probability of being in cluster C1:FT-Perm and in cluster C3:FT-Self rather than in cluster C4:Jless. Excluding the different types of deviant sequences (in red, blue, and green) did not change these conclusions in terms of significance, although the magnitude of the coefficients was sensitive to the exclusion of cluster-dissimilar sequences. Note that, for the interaction between maternal employment and geographical macro-area, both the magnitude and the significance of coefficients were sensitive to the exclusion of the deviant sequences. Specifically, compared to women in the Centre (reference category), daughters of employed mothers in the North-West (NW:M.Empl) and in the South (S:M.Empl) were only less likely to be in C3:FT-Self versus C4:Jless when structurally peculiar sequences were excluded from the sample (confidence intervals in red and in green in Fig. 2). On the contrary, for the daughters of employed mothers, for those in the North-West (NW:M.Empl) compared to those in the Centre, the difference in the probability of being in C1:FT-Perm versus C4:Jless was no longer significant when structurally peculiar sequences were excluded from the sample. In both cases, we see changes in the magnitude of the coefficients: in the first case from − 0.5969 to − 1.0436 (NW:M.Empl) and from − 0.5219 to − 1.4220 (S:M.Empl), in the second case from − 0.4247 to − 0.2842 (tables with the regression coefficients available from the authors). These results are particularly interesting given that removing the deviant sequences could lower the coefficients’ significance due to the reduced sample size; thus, in this case, deviant sequences obscured the direction and size of the effects. For researchers seeking to gather some insights about the mechanism underlying these findings, a first step would involve contrasting the baseline characteristics of the women who had regular versus (different types of) deviant sequences. Figure 7 in the Appendix﻿ reports the covariate distributions for the detailed set of types and sub-types identified using our procedures.

## Conclusions

SA is increasingly employed in different fields to describe longitudinal processes represented as sequences of categorical states. Many applications aim at simplifying the complexity of large sets of individual sequences and use cluster analysis to identify *types* representing different empirical realisations of the studied temporal process. Evaluating within-cluster homogeneity is crucial in SA to accurately describe the types. To address this issue, we introduced criteria to identify *deviant* sequences whose characteristics differ from the type process summarised by their cluster. We also introduce graphic tools to describe and contrast the features of regular and deviant cases, thus enhancing the description of the data structure. Besides allowing a more detailed analysis of clusters’ internal composition, our proposals enable an efficient identification and qualification of peculiar sequences that might be interesting in their own right from a substantive and theoretical point of view. As shown by our illustrative application, this cannot necessarily be accomplished by increasing the number of clusters, because clustering algorithms typically prioritise the identification of the most common patterns in data.

We elaborate on the possible role of deviant sequences in the (very typical) case when the relation between sequences and covariates is analysed using cluster membership as a response variable in a regression framework. In such cases, sequences in the same cluster are assumed to be fully consistent with the cluster type. Nonetheless, cases with deviant sequences might also differ from the other sequences in their clusters with respect to the covariate levels, especially when a strong association exists between sequences and covariates. This could influence and bias regression results. The role of deviant cases in determining the significance and magnitude of regression coefficients is demonstrated by our motivating application, focused on the link between maternal employment and daughters’ employment trajectories of Italian women across geographical areas. Our results suggest that maternal employment has an effect over and above parental education on the probability of daughters’ permanent employment over the life course. However, the significance of the interaction between maternal employment and geographical macro-area—which reflects different opportunities in the local labour market for female employment in general and for mother’s employment in particular—is sensitive to the exclusion of the deviant sequences from the clusters—and especially of structurally peculiar sequences. Thus, such employment trajectories are different from their cluster’s core, both in their structural characteristics (e.g., the states occurring over time) and the baseline characteristics of the individuals experiencing them. To conclude, researchers should carefully consider within-cluster homogeneity, the characteristics of regular and deviant sequences, and the possible role of the latter in subsequent analytical steps.

## Appendix

### A. List of all the Variables Employed in the Multinomial Model

**T﻿able 1:** Distribution of the covariates included in the multinomial logistic regression models

Variables’ levels	%
*Macro-area of residence (at interview)*	
North-East (R.NE)	21.6
North-West (R.NW)	21.9
Centre (R.C)	17.1
South (R.S)	28.9
Islands (R.I)	10.5
*Cohort*	
1959–1964 (C.59–64)	37.6
1965–1969 (C.65–69)	32.7
1970–1974 (C.70–75)	29.7
*Working status of the mother when the respondent was 15*	
Not working (M.Unemp)	61.3
Working (M.Emp)	38.7
*Highest parental education level*	
None (P.None)	9.5
Primary education (P.Prim)	49.3
At least lower-secondary education (P.LowSec+)	41.2
*At least one parent with tertiary education level*	
No (P.NTert)	96.0
Yes (P.Tert)	4.0
*N*	4323

*Source*: Multi-purpose Survey on Households: Families and Social Subjects 2009

### B. Analysis of Partitions Extracted from the Entire Set of Data

Figure 3 reports the quality of partitions with different numbers of clusters extracted using PAM and Ward’s hierarchical algorithms, with the former performing better. The 4-clusters solution is optimal with respect to different criteria: it maximises the Point biserial correlation and the average Silhouette coefficient, and—together with the 6-clusters solution—it minimises the Hubert’s C coefficient and maximises the Hubert’s Gamma index. Therefore, this could be a solution reasonably selected based on the considered indicators. Focusing on higher-order partitions, 6 and 9 clusters could be taken into account, as they are local optimisers.

In the Research Note, attention is focused on the 4-clusters partition. For the sake of completeness, Fig. 4 reports the index plots describing the 9-clusters partition. Increasing the number of clusters from 4 to 9 allows identifying one cluster including women whose trajectories are dominated by FT-Temp (C5), and one cluster (C6) including most of the women who studied for a relatively longer period—even if the evolution of the career after studying is highly heterogeneous, since these women entered the labour market with different types of contracts or remained unemployed. However, this more detailed partition does not allow isolating deviant sequences in separate clusters and is substantially devoted to offer a more accurate description of the differences among sequences dominated by the most frequent states, namely FT-Perm and JLess. This is reasonable, because of the relevance of these trajectories in data. Nonetheless, these results show that increasing the number of clusters does not guarantee the isolation and identification of structurally peculiar patterns. Similar considerations hold even when the number of clusters is further increased to 15 (see Fig. 5): the obtained partitions offer a more accurate description of the trajectories characterised by dominant states.

### C. A Detailed Analysis of the Deviant Sequences

To explore the characteristics of the sequences flagged as deviant based on the 4-clusters partition in details, we partitioned deviant sequences into 11 clusters (using PAM algorithm; the number of clusters was selected based on the same quality statistics considered in Fig. 3, results available from the authors). Figure﻿ 6 reports the index plots of the sequences identified as regular based on the 4-clusters partition (R1–R4) and of the 11 clusters of deviant sequences (D1–D11).

Some of the deviant clusters (e.g., D2 and D3) remain highly heterogeneous, even if the characteristics of the sequences allocated to them are more clearly distinguishable. In addition, very small clusters are identified including women whose work careers are dominated by less frequent employment statuses, such as DSelf (FT or PT, D6 and D9) or PT-Self (D8). Also, the trajectories characterised by a long initial track in Education are split into two clusters (D1 and D11) characterised by different final tracks. For the sake of completeness, we remark that this partition into 15-clusters solution is highly different from the 15-clusters partition extracted based on the entire set of data (Fig. 5). Indeed, the latter partition, which incidentally is not recommended by any criterion in Fig. 3, substantially offers a more detailed description of the trajectories dominated by the most frequent states, namely FT-Perm and JLess.

Table 2 reports the cross-tabulation of the 4-clusters (C1–C4) extracted from the entire data set and the 15 clusters of regular (R1–R4) and deviant (D1–D11) sequences. Note how the different deviant clusters spread across the original 4-clusters. For example, cluster D2.JLess → FT-Perm and D10. FT-Perm → JLess are included both in cluster C1:FT-Perm and in cluster C4:JLess. As discussed in the Research Note, the effect of excluding the deviant cases from the clusters depends on the characteristics of the deviant sequences, that is on their values on the covariates included in the model. If deviant sequences have covariates’ characteristics different from those of regular cases, their presence in the clusters might mask or at least partially compensate the relevance of some covariates, and their exclusion will lead to an increase of the effects’ magnitude and/or significance. In contrast, if deviant sequences are similar to the core sequences in the baseline characteristics, one can expect that excluding the former leads to a decrease in the effects’ magnitude and/or significance due to the sample-size effect or to the fact that the significant association between the cluster and the covariate was driven by the fact that the deviant sequences had extreme levels on the covariate. The understanding of the mechanisms leading to the observed changes in magnitude and significance in each empirical case requires not only an analysis of the covariates’ levels of deviant sub-types but also of the possible differences in covariates levels across “similar” deviant sequences assigned to different clusters.

**Table 2:** Cross-tabulation of the original 4-clusters partition extracted from the entire set of data and of the 15-clusters partition obtained by separating the regular cases in the 4 original clusters and the deviant sequences grouped into 11 clusters

Clusters of regular and deviant sequences	4-clusters extracted from the entire sample			
	C1:FT-Perm	C2:PT-Perm	C3:FT-Self	C4:Jless
R1:FT-Perm	1294			
R2:PT-Perm		206		
R3:FT-Self			203	
R4:Jless				1622
D1.LongEdu → FT-Perm	104	2	12	
D2.JLess → FT-Perm	47	2	3	63
D3.FT-Perm → PT-Perm	29	39	1	1
D4.FT-Perm → FT-Self	9	1	60	
D5.FT-Temp	22	8	45	81
D6.FT-DSelf	11	6	22	35
D7.PT-Temp	9		16	48
D8.PT-Self	3	2	10	10
D9.PT-DSelf	6	3	8	14
D10.FT-Perm → JLess	68	1	2	65
D11.LongEdu → JLess	1	12	34	83

*Source*: Multi-purpose Survey on Household and Social Subjects 2009

For the sake of illustration, ﻿Fig. 7 reports the distributions of the (categorical) covariates used in the regression model presented in the Research Note conditioned to the clusters of regular and deviant cases. Combining evidence from Table 2 and Fig. 7, we focus on the distribution across clusters of regular and deviant sequences of the variable accounting for the interaction between region of residence and the mother’s occupational position when the respondent was 15 years-old:

- Cluster C1: compared to regular sequences in cluster R1, the sequences flagged as deviant and reallocated to cluster D1 have a higher prevalence of employed mothers for almost all regions of residence, while those reallocated to cluster D10 have a higher prevalence of unemployed mothers from respondents in the North-Eastern and Centre regions.
- Cluster C2: compared to regular sequences in cluster R2, the sequences flagged as deviant and reallocated to cluster D3 presents fewer mothers in unemployment of respondents in the North-Western regions and more unemployed mothers of respondents in North-Eastern regions.
- Cluster C3: compared to regular sequences in cluster R3, the sequences flagged as deviant and reallocated to cluster D4 present more unemployed mothers of respondents in the North-Western and Centre regions, and fewer employed mothers of respondents in Centre regions, while those reallocated to cluster D5 have a higher prevalence of unemployed mothers of respondents in the North-Western regions and more employed mothers of responders in the Southern regions.
- Cluster C4: compared to regular sequences in cluster R4, the sequences flagged as deviant and reallocated to clusters D2, D5, and D11 show a much lower prevalence of unemployed mothers of respondents in the Southern regions.

## Footnotes

1 Some authors (Piccarreta & Studer, 2019; Jalovaara & Fasang, 2020) use the silhouette coefficients to identify sequences weakly related to their cluster and propose to drop sequences with negative or very low silhouette values before using the clusters in a regression model. The silhouette coefficient is based on the comparison between the average distance of a case to the cases in its cluster and the average distance to the cases in the closest cluster. Unlike the procedure introduced here, the silhouette coefficients allow us to identify sequences *between* clusters but do not detect cases distant from their cluster that are not close to other clusters.

2 Information is available on the starting and ending month of each employment, joblessness, or education spell. In the few cases of overlapping, we prioritized employment over joblessness and education, and education over joblessness.

3 In Italy, the deregulation reforms of the early 1990s introduced para-subordinate collaboration, under which workers are self-employed in legal terms but they are often ‘economically dependent’ on an employer because their activity relies only upon the clients provided by the employer. The category Dependent Self-employment therefore includes project-based work, occasional work, being a member of a cooperative: all these arrangements are not regulated by subordinate contracts.

4 All elaborations were obtained using the software R version 4.2.2 (R Core Team, 2022): packages *TraMineR* and *TraMineRExtra* (Gabadinho et al., 2011) *WeightedClusters* (Studer, 2013), *seriation* (Hahsler et al., 2008), and *nnet* (Venables & Ripley, 2002). The code to identify and plot deviant sequences is available at https://github.com/raffaellapiccarreta/.
